# A unique tripartite collision tumor of the esophagus: a case report and literature review

**DOI:** 10.3389/fonc.2024.1497154

**Published:** 2024-11-26

**Authors:** Shuai Luo, Xiaoxue Tian, Ting Xu, Jinjing Wang

**Affiliations:** Department of Pathology, Affiliated Hospital of Zunyi Medical University, Zunyi, Guizhou, China

**Keywords:** collision tumor, carcinoma, sarcoma, esophagus, adenoid cystic carcinoma, diagnosis

## Abstract

**Background:**

The coexistence of two or more distinct neoplasms within the same anatomical site characterizes collision tumors. While the presence of dual tumors is frequently observed in esophageal cases, the simultaneous occurrence of three distinct tumor types is extremely rare, posing significant challenges for pathological evaluation and diagnosis. Surgical resection remains the primary treatment, with generally favorable outcomes.

**Case presentation:**

A 58-year-old male with a two-month history of progressively worsening dysphagia over the past 10 days underwent a gastrointestinal barium meal examination, which revealed an irregular filling defect measuring approximately 89×50 mm in the mid-thoracic esophagus. Subsequent gastroscopic biopsy confirmed undifferentiated pleomorphic sarcoma in the mid-esophageal tissue. As the dysphagia advanced, a partial esophagectomy with lymph node dissection was performed. Postoperative pathology revealed a composite tumor consisting of adenoid cystic carcinoma, undifferentiated pleomorphic sarcoma, and focal squamous cell carcinoma. Squamous cell carcinoma metastasis was identified in one lymph node. No adjuvant therapies, such as chemotherapy, radiotherapy, targeted therapy, or immunotherapy, were administered following surgery. The patient had been under monitoring for 101 months, with no signs of recurrence or metastasis.

**Conclusion:**

This case represents the first documented instance of a tripartite collision tumor in the esophagus, composed of undifferentiated pleomorphic sarcoma, squamous cell carcinoma, and adenoid cystic carcinoma, with clear histological distinction. A thorough review of the literature was performed to summarize clinicopathological features. Surgical resection leads to a favorable prognosis. Tumors containing both carcinomatous and sarcomatous elements tend to have a more favorable prognosis compared to those composed entirely of carcinomatous tissue, providing valuable insights for future diagnostic and therapeutic strategies.

## Background

A collision tumor comprises two independent cell populations of different origins that coexist in juxtaposition to one another within the same tissue or organ, without any intermixing (1). The underlying mechanisms responsible for their development are still not well clarified. Genetic analyses indicate that these neoplasms may arise from a shared malignant progenitor cell that subsequently differentiates into two distinct lineages, each maintaining its own malignancy (1, 2).

In 1972, Spagnolo and Heenan formulated diagnostic criteria for collision tumors as follows: (1) The tumor must exhibit two distinct components, each with different tissue structures and origins; (2) A clear demarcation between the components should be identified, though the presence of an intermingling region does not preclude the dual origin; (3) Various transitional patterns may be noted within the collision zone, but metastatic tumors must be definitively excluded before diagnosis is established (3). Collision tumors have been reported in anatomical locations such as the sellar region, esophagus, cardia, liver, uterus, ovaries, adrenal glands, and other organs. Histologically, collision tumors often involve both epithelial and mesenchymal origins, requiring careful differentiation from other neoplasms like carcinosarcomas, which are biphasic tumors containing epithelial, predominantly squamous cell carcinoma, and spindle cell components; composite tumors, which show mixed histological patterns within a single tumor; or tumor-to-tumor metastasis. Collision tumors are exceptionally uncommon, and the current literature is largely confined to case reports, leaving their biological behavior poorly characterized.

The report discusses a case of an esophageal tripartite collision tumor comprising undifferentiated pleomorphic sarcoma, squamous cell carcinoma, and adenoid cystic carcinoma, with favorable postoperative outcomes. Tumors exhibiting both carcinomatous and sarcomatous elements generally display a more promising prognosis compared to those containing solely carcinomatous components. Improving diagnostic precision in pathology is essential to inform clinical decision-making and optimize treatment strategies.

## Case presentation

A 58-year-old male with a 40-year history of smoking (20 cigarettes/day) and alcohol consumption (250 grams/day) presented with progressive dysphagia over the past two months. He worked in a coal mine for more than a decade, with no family history of malignancy. His medical history was otherwise unremarkable, with no prior infections such as hepatitis, tuberculosis, or typhoid fever. He also had no history of surgery, trauma, blood transfusion, or known allergies.

Two months earlier, he experienced dysphagia of unknown origin, accompanied by acid reflux, epigastric discomfort, and abdominal bloating, without hematemesis, melena, cough, or sputum production. There were no symptoms of cold intolerance, fever, chest tightness, or palpitations, and he initially dismissed the issue. However, ten days ago, his symptoms worsened, now including diarrhea, prompting admission to a local county hospital. Oral medication became intolerable due to his difficulty swallowing. Subsequent gastroscopy revealed food residue obstructing the esophageal lumen, leading to a diagnosis of esophageal stenosis and referral to our department for further evaluation and management. Despite these symptoms, his general condition, including mental status, diet, sleep, and weight, remained stable, though occasional diarrhea persisted. Urination was normal.

On physical examination, his vital signs were as follows: T 36.5°C, P 90 beats/min, R 20 breaths/min, BP 121/75 mmHg. The abdomen was flat and soft, with no visible gastrointestinal distension or peristaltic waves. Mild tenderness was noted near the xiphoid process, but there was no rebound tenderness or muscle rigidity. The liver and spleen were not palpable, Murphy’s sign was negative, and there was no pain on percussion in the liver or kidney regions. Shifting dullness was negative.

Laboratory tests revealed the following: serum sodium 132.1 mmol/L, aspartate aminotransferase 76 U/L, and total bilirubin 14.9 μmol/L. Blood lipid profile, renal function, blood glucose, and coagulation parameters were within normal limits. Electrocardiography showed no abnormalities.

**Figure 1:**
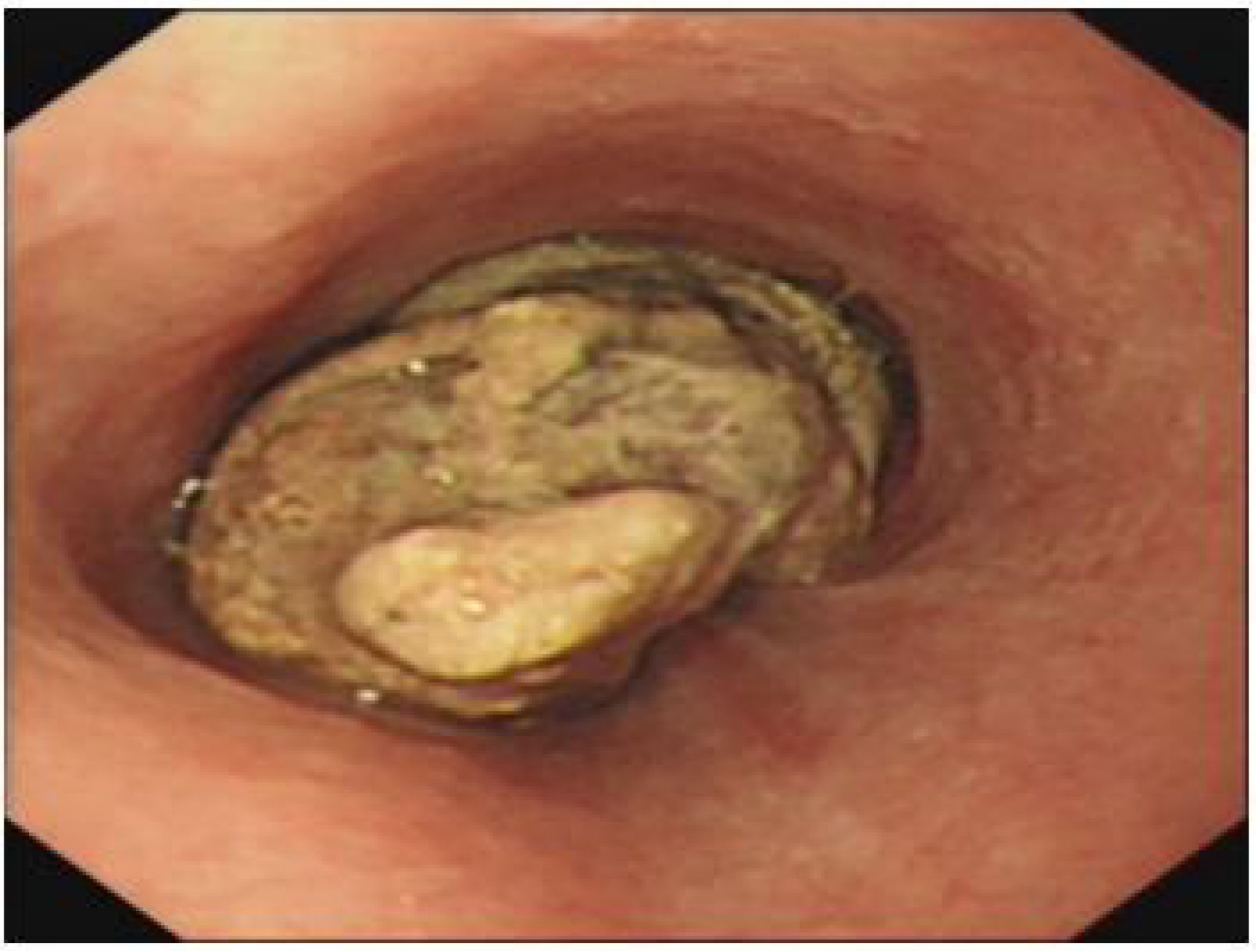
Gastroscopy identified a mass extending 28-32 cm from the incisor, occupying two-thirds of the esophageal lumen.

Chest computed tomography (CT) revealed emphysema, bilateral pulmonary bullae, and interstitial lung lesions characterized by extensive fibrosis, proliferation, and calcification across both lungs. Endoscopic evaluation was advised to further assess the irregularities in the mid-thoracic esophagus. A barium swallow study indicated a substantial filling defect, approximately 89×50 mm in size, located below the impression of the aortic arch. This defect, with a roughened inner surface, resulted in significant luminal constriction and impaired peristalsis. Gastroscopy confirmed the presence of a mass, 28-32 cm from the incisors, occupying two-thirds of the esophageal lumen. The mass exhibited a firm texture, with visible food remnants, surface exudates, spontaneous and contact bleeding (**Figure 1**). A biopsy was obtained for histopathological evaluation.

**Figure 2:**
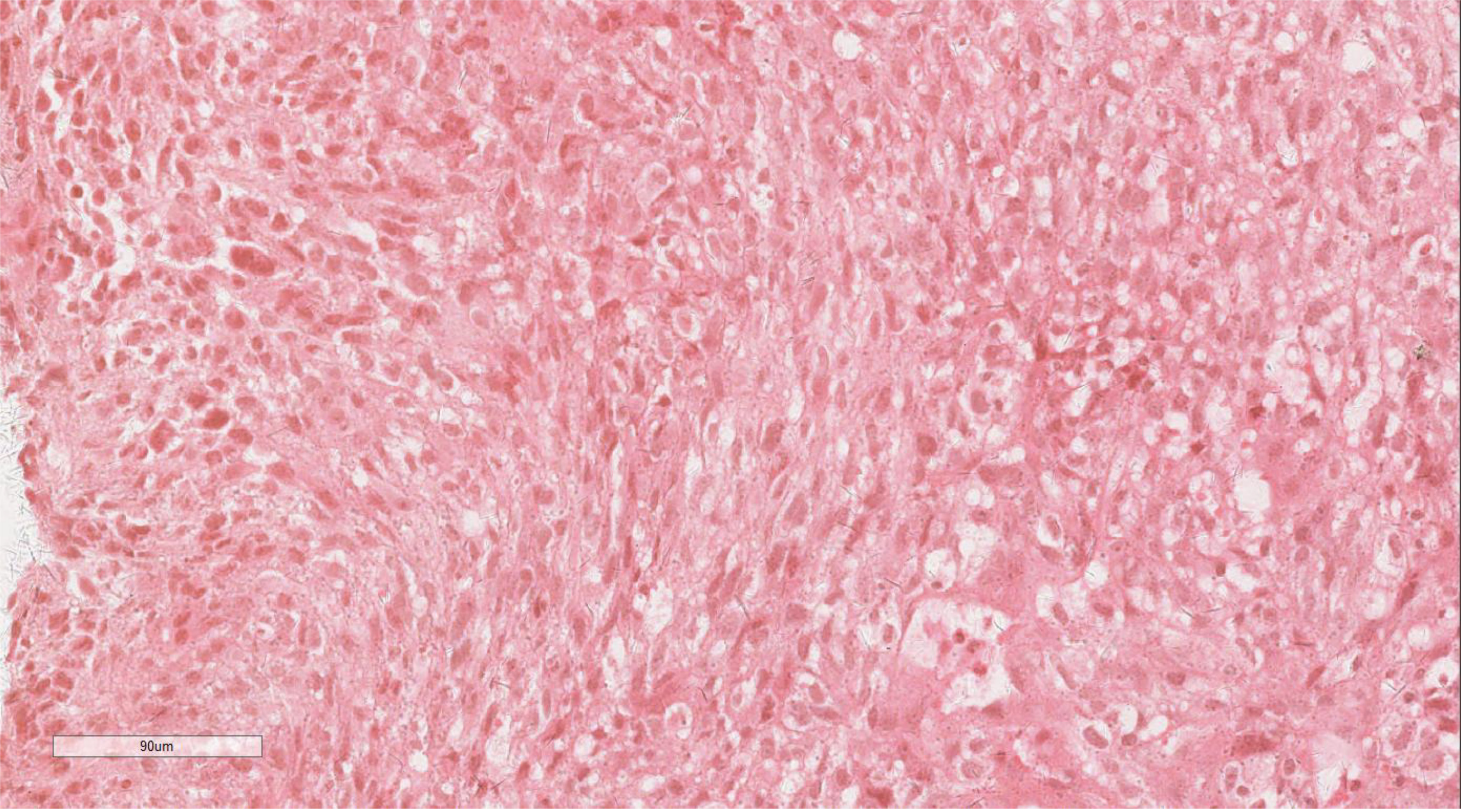
Histopathologic assay of the biopsy revealed a proliferation of oval or spindle cells. H&E ×220.

Histopathological analysis of the biopsy identified a proliferation of oval and spindle-shaped cells arranged in either interwoven or diffuse nest-like formations, displaying marked cellular atypia, necrosis, sporadic mitotic activity, and scattered multinucleated giant cells (**Figure 2**). Immunohistochemical staining revealed hyperplastic spindle cells expressing Vimentin, partial LCA positivity, and the following marker profile: CD34 (-), CD56 (+/-), CD68 (+), CK (-), CK5/6 (-), CK7 (-), CHB (-), Desmin (showing a limited number of focal positive cells), EMA (+/-), HMB45 (-), P40 (-), p63 (-), S100 (-), SMA (-), CD117 (-), and Dog-1 (-). The pathological diagnosis strongly suggested a malignancy in the middle esophagus, with differential diagnoses including malignant fibrous histiocytoma or undifferentiated pleomorphic sarcoma.

An esophagectomy with systemic lymphadenectomy was conducted to alleviate worsening dysphagia. Intraoperative assessment identified a mid-thoracic tumor approximately 8.5 cm in length and 5 cm in maximum transverse diameter. The mediastinal pleura near the esophagus was dissected without definitive signs of tumor infiltration. Gross pathological evaluation encompassed a 27 cm section of the esophagus. A polypoid lesion, located 4.5 cm from one margin and 12 cm from the other, protruded into the lumen. The lesion, measuring 8.0 × 5.0 × 1.0 cm, exhibited an irregular surface with remnants of food, and its cut surface was gray-red to gray-white with a soft texture. Additionally, 17 lymph nodes were retrieved, with sizes ranging from 0.2 cm to 1.7 cm.

**Figure 3:**
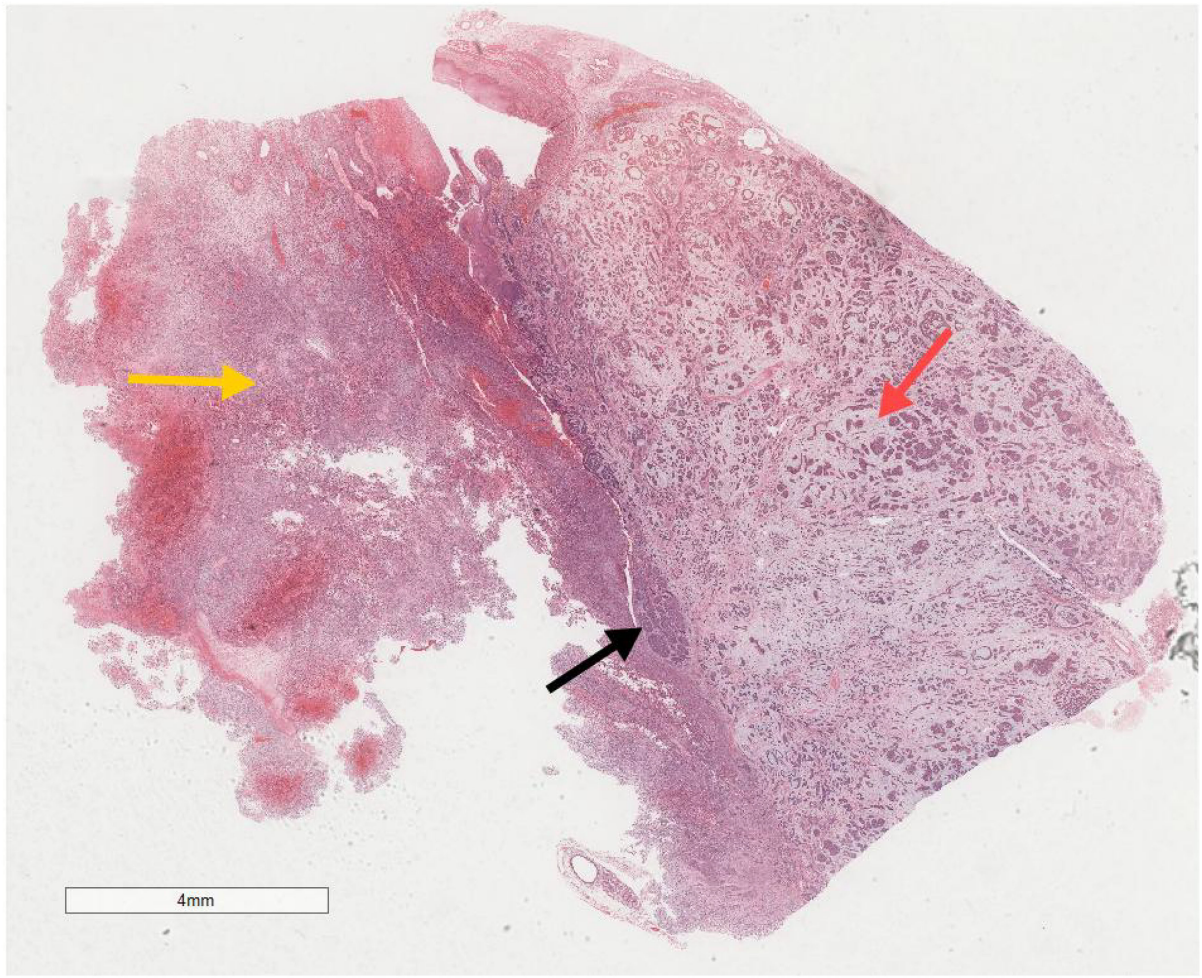
Microscopic analysis identified well-defined boundaries between the three tumor types: undifferentiated pleomorphic sarcoma (40%, Yellow arrow), squamous cell carcinoma (5%, Black arrow), and adenoid cystic carcinoma (55%, Red arrow). H&E ×5.

**Figure 4:**
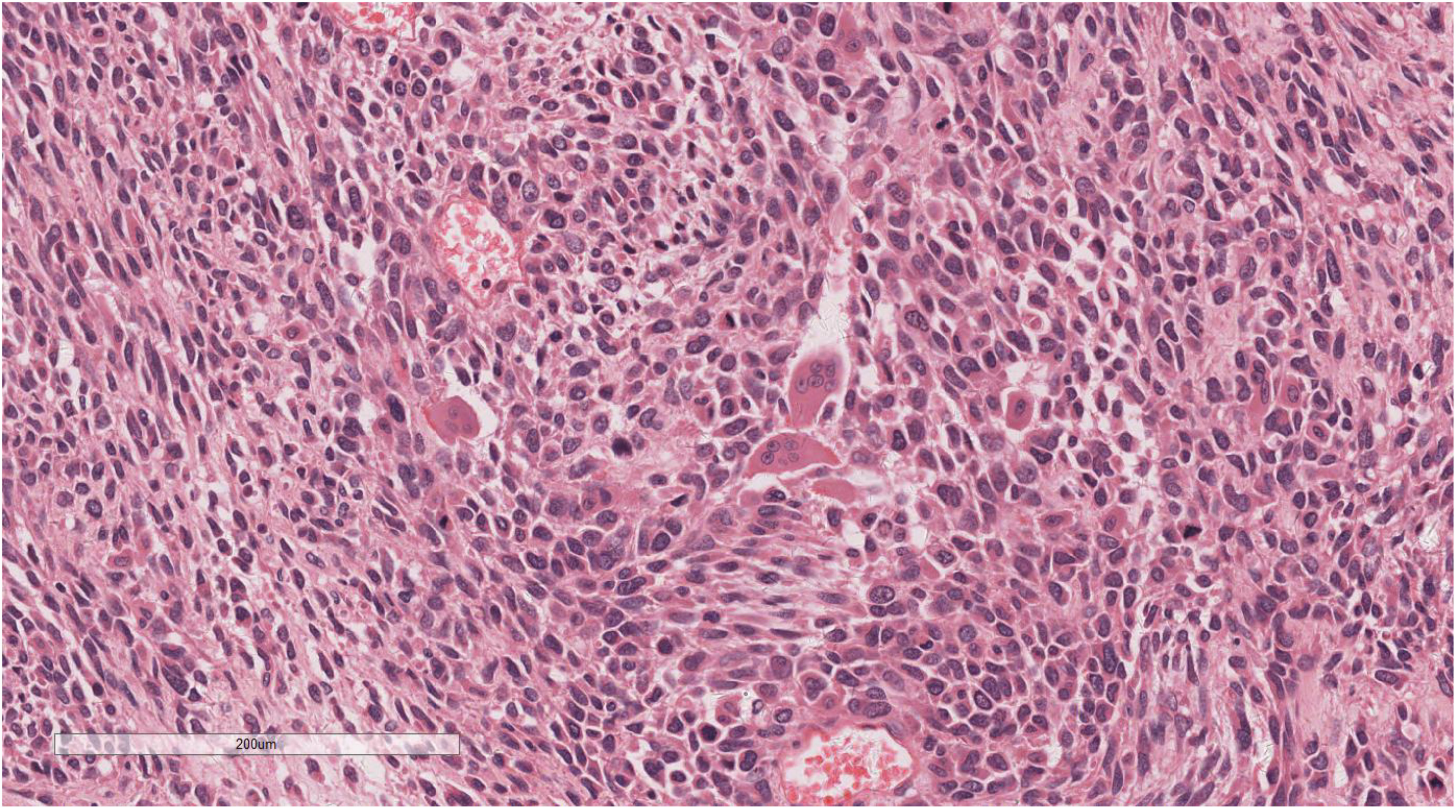
In Area 1, components of undifferentiated pleomorphic sarcoma. H&E ×200.

**Figure 5:**
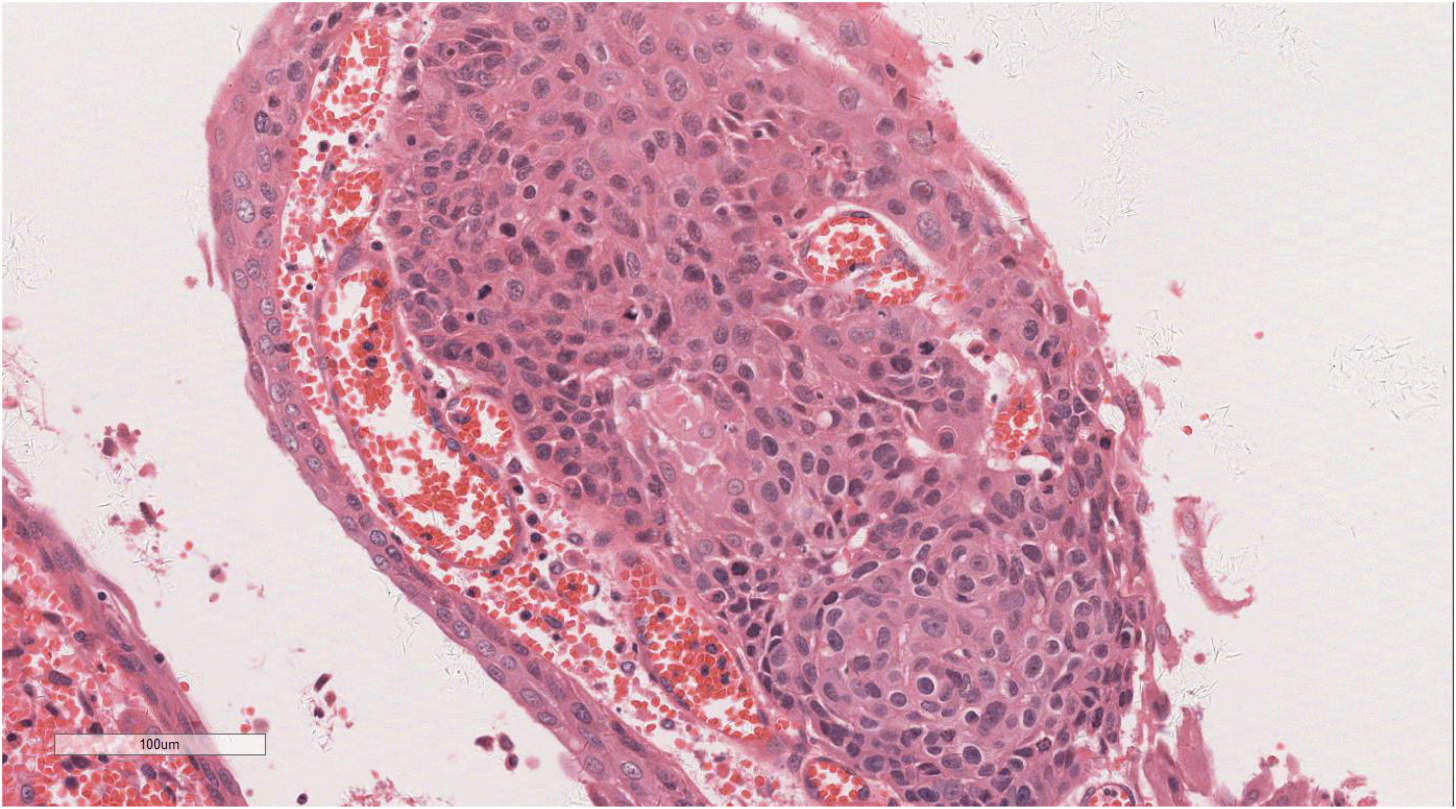
In Area 2, components of squamous cell carcinoma. H&E ×200.

**Figure 6:**
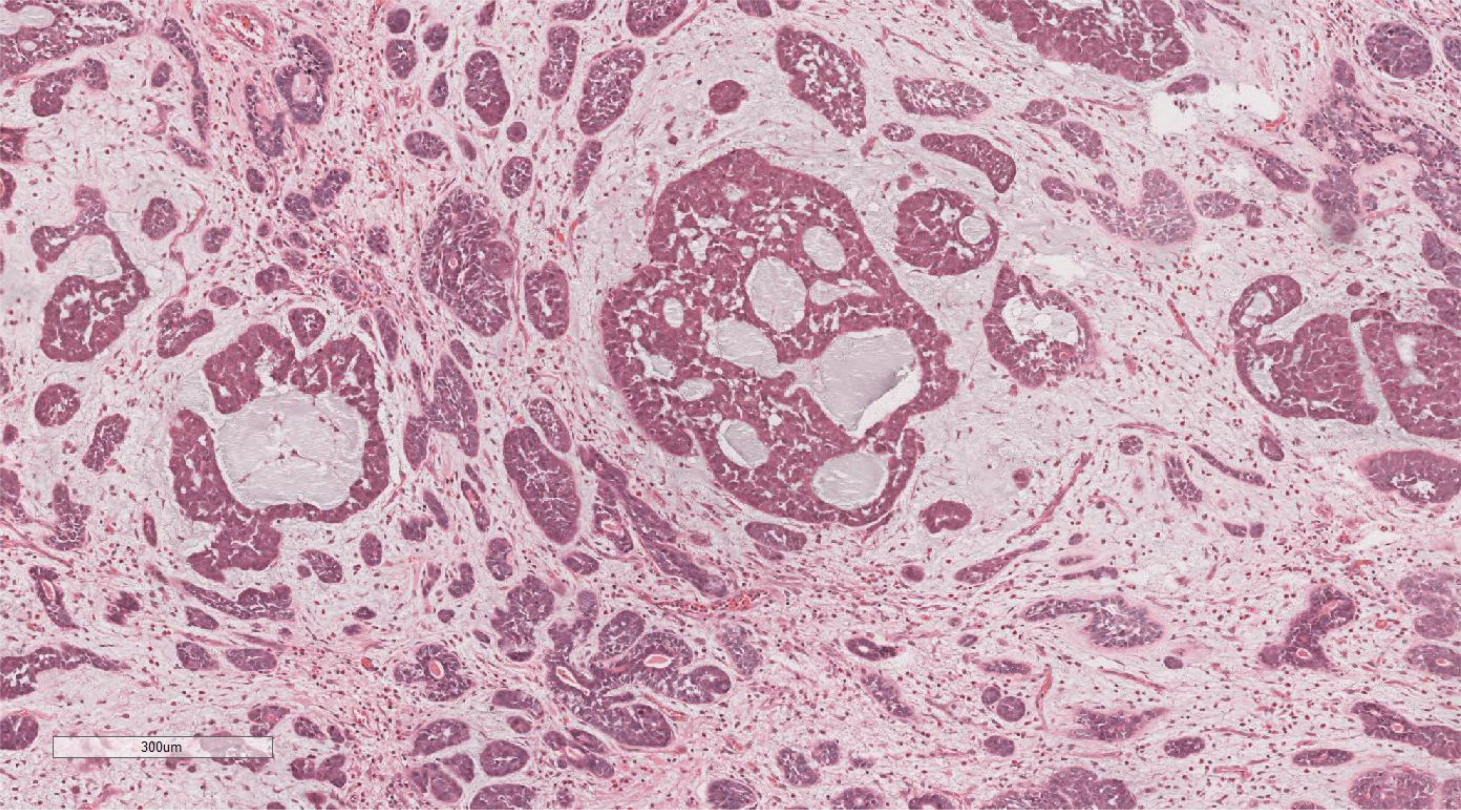
In Area 3, components of adenoid cystic carcinoma. H&E ×70.

Microscopic examination delineated distinct borders among the three tumor types: undifferentiated pleomorphic sarcoma (40%), squamous cell carcinoma (5%), and adenoid cystic carcinoma (55%) (**Figure 3**). In Area 1, associated with undifferentiated pleomorphic sarcoma (**Figure 4**), the tumor presented with high cellular density and a characteristic storiform architecture. Cellular morphology demonstrated significant pleomorphism, including spindle, short spindle, round, oval, and giant forms, with nuclei showing enlargement, hyperchromasia, prominent nucleoli, and frequent mitotic activity. The stroma exhibited extensive vascular proliferation and myxoid degeneration, along with necrotic and hemorrhagic regions. Area 2, corresponding to squamous cell carcinoma (**Figure 5**), revealed confinement to the mucosal layer with superficial spread. Tumor cells were arranged in sheets, displaying pale-staining nuclei, prominent nucleoli, and an absence of mitotic figures, alongside intercellular bridges and incomplete keratinization. In Area 3 (adenoid cystic carcinoma) (**Figure 6**), the tumor infiltrated the esophageal muscle layer with well-defined borders, extending into the overlying squamous epithelium. This region consisted of glandular epithelial and myoepithelial/basaloid cells, primarily organized in cribriform, tubular, and focal solid nest patterns. Within the cribriform areas, both true and pseudoglandular cavities were observed, containing eosinophilic secretions in the true cavities and mucus-like substances in the pseudoglandular counterparts. True glandular luminal cells displayed a cuboidal morphology, with eosinophilic cytoplasm, round nuclei, and small nucleoli, while pseudoglandular luminal cells, defined by a basaloid phenotype, appeared small, round, and translucent, also with small nucleoli. The tubular structure comprised 2-3 cellular layers, featuring ductal cells internally and myoepithelial cells externally. The lumen was filled with pink-staining mucus. Within the solid component, tumor cells arranged in compact nests presented large cellular and nuclear dimensions, with vacuolated nuclei, discernible nucleoli, and mitotic activity.

**Figure 7:**
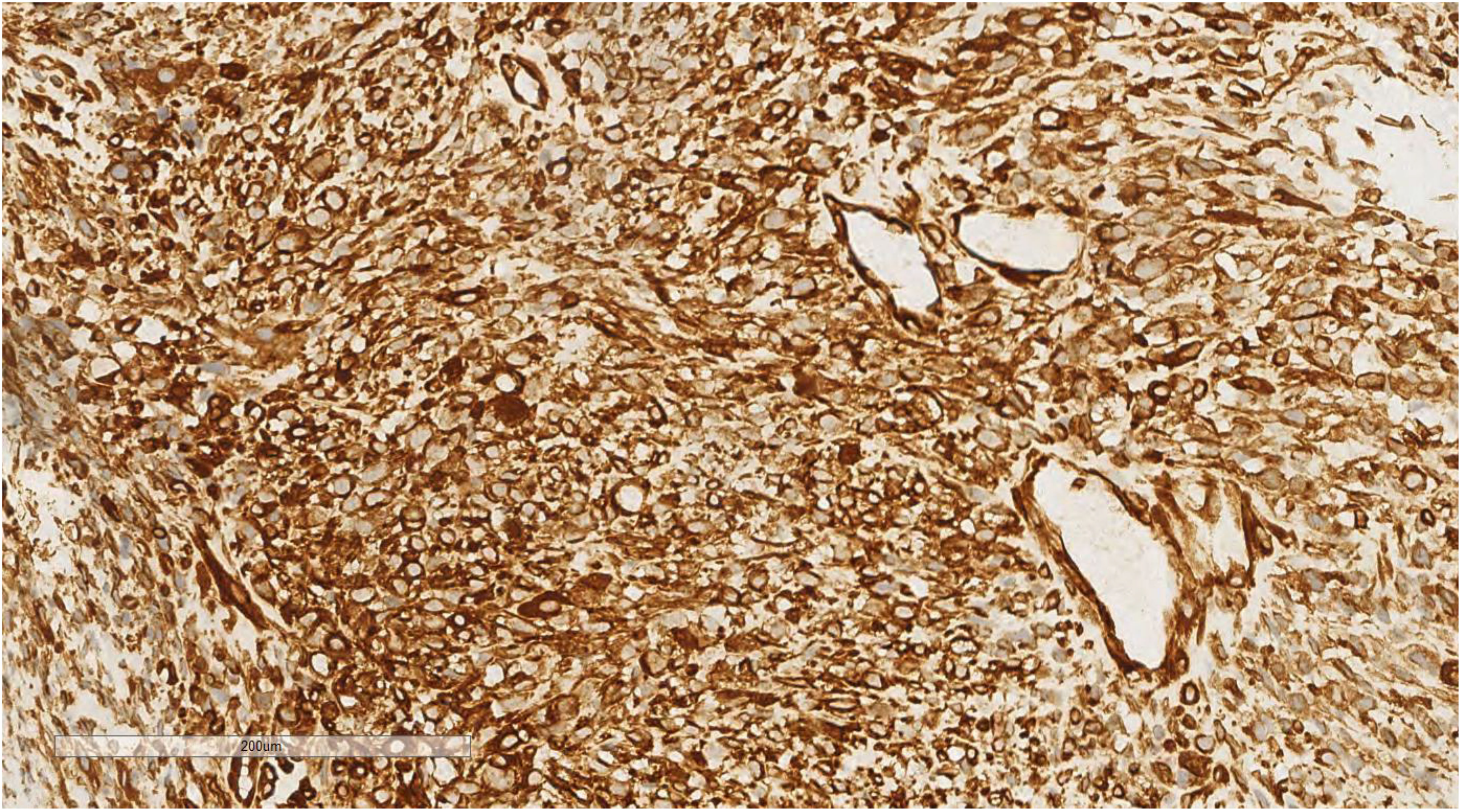
Immunohistochemical analysis of the undifferentiated pleomorphic sarcoma component revealed positivity for Vimentin. EnVision ×200.

**Figure 8:**
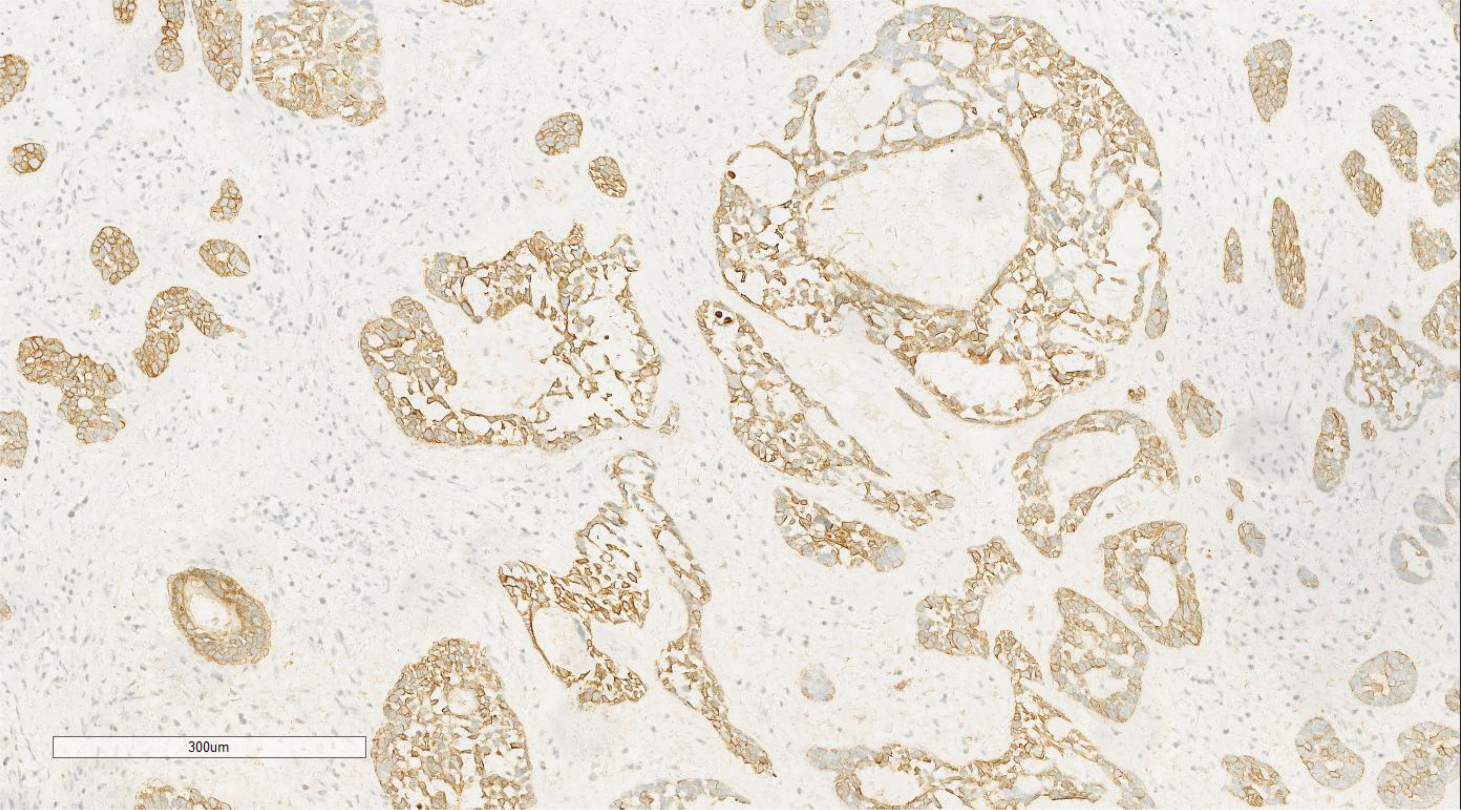
Immunohistochemical analysis of the squamous cell carcinoma component showed positivity for CK. EnVision ×100.

**Figure 9:**
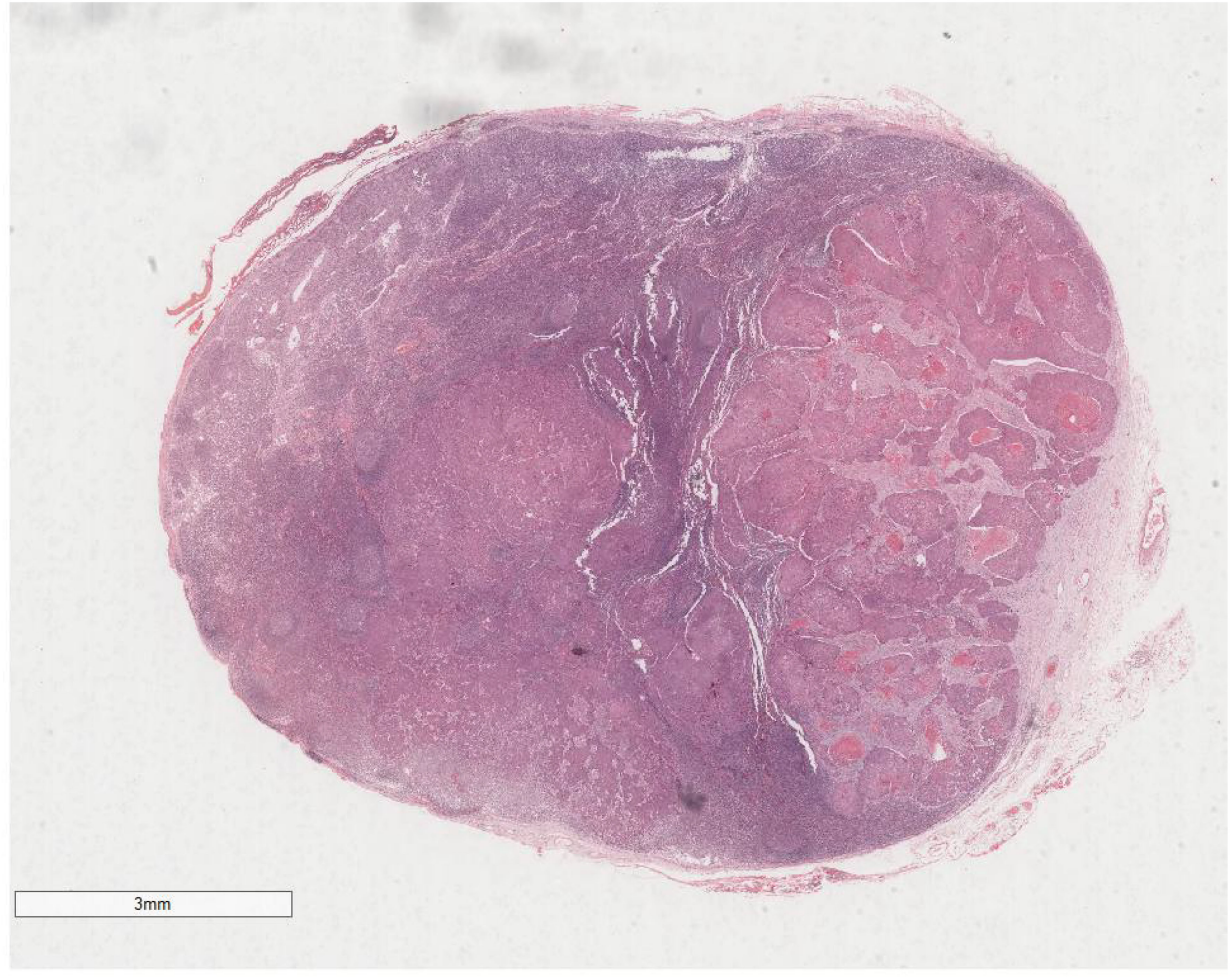
Metastasis of squamous cell carcinoma in one lymph node. H&E ×7.

Immunohistochemical analysis of the undifferentiated pleomorphic sarcoma component demonstrated positivity for Vimentin (**Figure 7**), CD68 (P-1), and Desmin in localized areas, with negative staining for CK, EMA, S100, SMA, MyoD1, Myoglobin, GFAP, Melan-A, and Lysozyme, and positive CD56 expression. The squamous cell carcinoma component exhibited positivity for CK (**Figure 8**), CK5/6, EMA, P40, p63, and focal positivity for CK7, Vimentin, and BerEP4, while markers such as S100, SMA, CD56, Syn, and CgA were negative. The adenoid cystic carcinoma component showed peripheral positivity for P63 and CK5/6 in basal cells, CD117, and EMA in glandular structures, with weak focal positivity for S100 and SMA, and negative Vimentin expression. Morphological and immunohistochemical evaluations classified the tumor as a collision tumor comprising undifferentiated pleomorphic sarcoma, squamous cell carcinoma, and adenoid cystic carcinoma. Among the 17 lymph nodes examined, metastasis of squamous cell carcinoma was detected in one node (**Figure 9**). Specifically, cancer metastasis (squamous cell carcinoma) was identified in one of two lymph nodes from the middle esophagus. No metastasis was observed in the upper para-esophageal (0/1), lower para-esophageal (0/2), left recurrent laryngeal nerve (0/4), right recurrent laryngeal nerve (0/2), left main bronchus (0/3), right main bronchus (0/3), subcarinal (0/3), or lymph nodes adjacent to the left gastric artery (0/3) and celiac trunk (0/3).

In conclusion, the patient’s treatment followed a comprehensive approach: post-admission examinations were promptly optimized, and thorough preoperative preparation was completed. Under general anesthesia, a combined thoracoscopic and laparoscopic procedure was performed, involving cervical, thoracic, and abdominal incisions for middle esophageal cancer resection, gastroesophageal cervical anastomosis, and systematic lymphadenectomy. Postoperatively, the patient received anti-inflammatory, expectorant, acid-suppressive therapy, along with intravenous and enteral nutrition support, resulting in satisfactory recovery. Digestive tract angiography reexamination confirmed a normal anastomosis, after which the patient was gradually reintroduced to food, underwent regular dressing changes, and had the drainage tube removed.

After 14 days of hospitalization, the patient demonstrated significant recovery, exhibiting stable vital signs and absence of symptoms such as chills, fever, nausea, vomiting, cough, sputum production, or dyspnea. Tolerance to a semi-liquid diet was reported without any discomfort, and the patient’s mental state remained positive. Physical examination revealed clear consciousness, stable respiration, and normal lung auscultation without dry or wet rales. The surgical incision showed no signs of infection, and a follow-up examination of the digestive system returned normal findings. The patient was subsequently discharged with a plan for routine follow-up assessments.

Following surgical intervention, the patient underwent a 101-month follow-up. Gastroscopy revealed the anastomosis located 18 cm from the incisor, with surrounding mucosal congestion and cord-like erosions. The cardia demonstrated proper functioning, with a clearly defined dentate line. Both gastric and duodenal mucosa appeared unremarkable. Gastroscopic findings included postoperative alterations of esophageal Ca, anastomotic stomatitis, and chronic atrophic gastritis (C-1). To date, no recurrence or metastasis has been observed, and the prognosis remains favorable.

## Discussion

Squamous cell carcinoma comprises nearly 90% of esophageal cancers, primarily involving the mid-esophagus. Adenoid cystic carcinoma, more commonly linked to salivary glands, appears rarely within the esophagus, with reported cases largely confined to its middle section. Undifferentiated pleomorphic sarcoma in the esophagus is exceedingly rare, mainly presenting in the middle and distal segments. Literature predominantly addresses esophageal collision tumors consisting of two separate neoplastic types, such as squamous cell carcinoma co-occurring with adenoid cystic carcinoma (4), leiomyoma (5), neuroendocrine carcinoma (6), or leiomyosarcoma (7). Reports also document instances of squamous cell carcinoma with small cell carcinoma (8). Documented occurrences of collision tumors involving three distinct neoplasms are exceptionally rare, with only six cases recorded to date. These include an esophageal collision tumor comprising small cell carcinoma, moderately differentiated adenocarcinoma, and signet ring cell carcinoma (9); three cases combining squamous cell carcinoma, adenocarcinoma, and small cell carcinoma (10); a unique instance with squamous cell carcinoma, ciliated adenocarcinoma, neuroendocrine carcinoma, and sarcoma (11); and another case featuring small cell carcinoma, adenocarcinoma, and squamous cell carcinoma (12). Furthermore, two cases have described esophageal collision tumors involving undifferentiated pleomorphic sarcoma (11, 13). This report presents a previously undocumented case of an esophageal collision tumor encompassing undifferentiated pleomorphic sarcoma, squamous cell carcinoma, and adenoid cystic carcinoma.

The sarcomatous component is located above the tumor, eroding the surface squamous epithelium while maintaining distinct boundaries. Adjacent squamous mucosa shows evidence of squamous cell carcinoma *in situ*. Beneath this, adenoid cystic carcinoma emerges as the predominant histological feature. The separation between these three components remains clearly demarcated. The tumor’s pathogenesis is not fully understood, and whether the observed growth pattern represents a typical developmental course remains uncertain. The polyclonal hypothesis suggests that distinct differentiation pathways arise from multiple stem cells. Thompson et al. (14), in a clonal analysis of six carcinosarcoma cases, demonstrated monoclonality in both carcinomatous and sarcomatous elements, supporting the theory of a single totipotent stem cell origin. Other studies (15) propose that the sarcomatous component originates from cancer cells via tumor metaplasia, consistent with the monoclonal hypothesis of carcinosarcoma differentiation, wherein a single totipotent stem cell gives rise to both epithelial and mesenchymal lineages. In the current case, well-defined tumor margins and immunohistochemical marker patterns favor the metaplastic origin theory. However, larger studies are required to substantiate these findings.

As detailed in **Table 1**, the three cases involved elderly male patients aged 58-64 years, with dysphagia as the predominant clinical symptom. All tumors were situated in the distal esophagus, with diameters ranging from 1.5 to 8 cm. Collision tumors present significant diagnostic challenges due to their lack of distinct clinical or imaging features. In this instance, diagnosis was initially established via puncture biopsy, though only one tumor component was identified, underscoring the inherent risk of incomplete or incorrect diagnoses with this method. A conclusive diagnosis necessitates thorough sampling from resected surgical specimens to ensure comprehensive assessment. Postoperative outcomes were favorable in all three cases. In a study (9), lymph node metastasis was detected at diagnosis, with the metastatic component confirmed as adenocarcinoma. Six months post-surgery and chemotherapy, multiple liver metastases developed. According to Reference 10, three patients experienced rapid tumor recurrence following esophagectomy, with all succumbing to the disease within 14 months; metastases primarily consisted of small cell carcinoma. In the case described in Reference 12, vertebral, hepatic, and cutaneous metastases were detected one month post-surgery, with the patient dying two months and nine days later. Collision tumors comprising both carcinomatous and sarcomatous components tend to show a more favorable prognosis than purely carcinomatous tumors. However, this conclusion is based on a single case report, and further large-scale studies are necessary for validation.

**Table 1:** Clinicopathological characteristics of esophageal collision tumor with undifferentiated sarcoma.

Case	Reference	Gender	Age	Location	Clinical Manifestation	Treatment Strategy	Tumor Component	Lymph Node Metastasis Component	Tumor Size (cm)	Prognosis	Follow-up duration (months)
1	Kanamoto A (11)	Male	58	Lower 1/3 of esophagus	Dysphagia	Surgery	Sarcoma, squamous cell carcinoma, ciliated adenocarcinoma, and neuroendocrine carcinoma	No	1.5×1.4×1.0	Death from myocardial infarction	33
2	Thompson L (14)	Male	64	Lower 1/3 of esophagus	Dysphagia	Surgery	Sarcoma, squamous cell carcinoma, adenocarcinoma, and neuroendocrine carcinoma	No	2.0×1.5×0.8	No recurrence or metastasis	12
	This case	Male	58	Lower 1/3 of esophagus	Dysphagia	Surgery	Squamous cell carcinoma, adenoid cystic carcinoma, and sarcoma	Squamous cell carcinoma	8.0×5.0×1.0	No recurrence or metastasis	101

## Conclusion

This study presents a rare case of a tripartite collision tumor in the esophagus, consisting of undifferentiated pleomorphic sarcoma, squamous cell carcinoma, and adenoid cystic carcinoma. A comprehensive literature review was performed to elucidate the clinicopathological characteristics of this tumor, providing essential information for diagnostic and therapeutic strategies.

## Data Availability

The original contributions presented in the study are included in the article/supplementary material. Further inquiries can be directed to the corresponding author.
